# Association between pesticide exposure and thyroid function: analysis of Chinese and NHANES databases

**DOI:** 10.3389/fpubh.2024.1378027

**Published:** 2024-06-12

**Authors:** Leiming Xu, Shengkai Yang, Longqing Wang, Jinxin Qiu, Hai Meng, Lulu Zhang, Wenwen Sun, Aifeng He

**Affiliations:** ^1^Binhai County People's Hospital Affiliated to Kangda College of Nanjing Medical University, Yancheng, Jiangsu, China; ^2^Department of Intensive Care Unit, Changzhou Maternity and Child Health Care Hospital, Changzhou Medical Center, Nanjing Medical University, Changzhou, China

**Keywords:** pesticides, thyroid function, Bayesian kernel machine regression, restricted cubic spline curve, NHANES database

## Abstract

**Background:**

Pesticides are widely used in agricultural activities. Although pesticide use is known to cause damage to the human body, its relationship with thyroid function remains unclear. Therefore, this study aimed to investigate the association between pesticide exposure and thyroid function.

**Methods:**

The Chinese database used included 60 patients with pyrethroid poisoning and 60 participants who underwent health checkups between June 2022 and June 2023. The NHANES database included 1,315 adults enrolled from 2007 to 2012. The assessed pesticide and their metabolites included 2,4-dichlorophenoxyacetic acid (2,4-D), 4-fluoro-3-phenoxybenzoic acid (4F3PB), para-nitrophenol (PN), 3-phenoxybenzoic acid (3P), and trans-dichlorovinyl-dimethylcyclopropane carboxylic acid (TDDC). The evaluated indicators of thyroid function were measured by the blood from the included population. The relationship between pesticide exposure and thyroid function indexes was investigated using linear regression, Bayesian kernel machine regression (BKMR), restricted cubic spline (RCS), and weighted quantile sum (WQS) models.

**Results:**

The Chinese data showed that pesticide exposure was negatively correlated with the thyroid function indicators FT4, TT4, TgAb, and TPOAb (all *p* < 0.05). The BKMR model analysis of the NHANES data showed that the metabolic mixture of multiple pesticides was negatively associated with FT4, TSH, and Tg, similar to the Chinese database findings. Additionally, linear regression analysis demonstrated positive correlations between 2,4-D and FT3 (*p* = 0.041) and 4F3PB and FT4 (*p* = 0.003), whereas negative associations were observed between 4F3PB and Tg (*p* = 0.001), 4F3PB and TgAb (*p* = 0.006), 3P and TgAB (*p* = 0.006), 3P and TPOAb (*p* = 0.03), PN and TSH (*p* = 0.003), PN and TT4 (*p* = 0.031), and TDDC and TPOAb (*p* < 0.001). RCS curves highlighted that most pesticide metabolites were negatively correlated with thyroid function indicators. Finally, WQS model analysis revealed significant differences in the weights of different pesticide metabolites on the thyroid function indexes.

**Conclusion:**

There is a significant negative correlation between pesticide metabolites and thyroid function indicators, and the influence weights of different pesticide metabolites on thyroid function indicators are significantly different. More research is needed to further validate the association between different pesticide metabolites and thyroid disease.

## Graphical abstract

WQS, weighted quantile sum; BKMR, Bayesian kernel machine regression; RCS, restricted cubic spline; 2,4-D, 2,4-dichlorophenoxyacetic acid; 4F3PB, 4-fluoro-3-phenoxybenzoic acid; 3P, 3-phenoxybenzoic acid; PN, para-nitrophenol; TDDC, trans-dichlorovinyl-dimethylcyclopropane carboxylic acid.

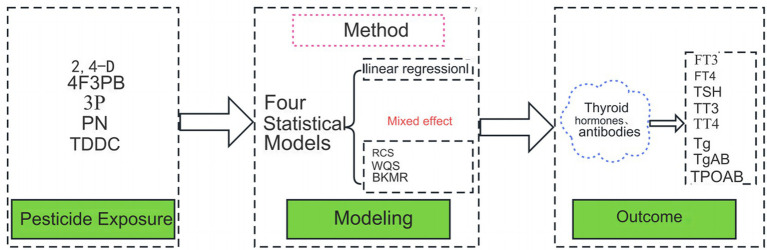

## 1 Introduction

Pesticides are the most widely used chemical compounds in agriculture, mainly for preventing and eliminating pests and reducing crop damage risk. Current studies suggest that numerous pesticides are more or less harmful to human health. For example, ethyl dithiocarbamate (EBDC) is an extensively applied pesticide worldwide that can cause hypothyroidism in rats, primarily manifesting as decreased FT4 and increased TSH levels (1, 2). Other research has identified the same effect of EBDC on human thyroid function, resulting in decreased thyroid function and serum TSH concentration among people exposed to EBDC compared to those not in contact with EBDC (3). Pyrethroid insecticides and herbicides are not new to the agricultural field, with pyrethroids observed to disrupt thyroid function by binding to hormone receptors due to their structural similarities with the receptors (4). Organophosphorus pesticides (OPs) are another pesticide class popularly employed in agricultural activities. Exposure to these OPs or multiple pesticide classes is associated with genotoxicity and adverse neurobehavioral outcome markers in exposed populations, especially children and farm workers (5). All these research findings highlight that the effects of various pesticides on human health cannot be ignored.

2,4-dichlorophenoxyacetic acid (2,4-D), also known as the “king of grass,” is a commonly utilized herbicide and synthetic plant hormone that controls weed growth by mimicking the effects of auxin. Studies have revealed a significant increase in hypothyroidism among people using 2,4-D pesticides (6). Moreover, Goldner et al. (7) identified a significant association between 2,4-D pesticide exposure and hypothyroidism in male pesticide users, whereas no such significant association was detected in the female population. Similar conclusions have been found recently, the United States Environmental Protection Agency’s (US EPA) Endocrine Disruptor Screening Program test, which examines potential interactions between 2,4-D and the estrogen, androgen, and thyroid pathways or steroid production, found no convincing evidence on the potential interaction between 2,4-D and estrogen. However, a potential interaction was indicated between 2,4-D and the androgen and thyroid pathways (8, 9). Animal studies have also demonstrated that rats subjected to different doses of 2,4-D exhibited varied effects on thyroid hormone levels as well as on the weight and pathology of the thyroid gland (10).

Para-nitrophenol (PN) and trans-dichlorovinyl-dimethylcyclopropane carboxylic acid (TDDC) are metabolites of the extensively employed OPs and insecticides. OPs are mainly used for controlling pests, such as mosquitoes and fleas, in agriculture, homes, and public places. The primary mechanism of OPs is to produce an insecticidal effect by acetylcholinesterase inhibition in the nervous system of pests. Researchers have determined that compared to the general male population, men exposed to OPs presented with significant changes in the Thyroid Stimulating Hormone (TSH) and other thyroid hormone levels (11). Consistent with the occupational and experimental study findings, OPs can significantly escalate hypothyroidism. The hyperthyroidism changes include increased TSH levels and a reduction, increase, or no significant changes in the T3 and/or T4 levels (12). According to the above findings, OPs and carbamate insecticides may inhibit brain cholinesterase activity by affecting the hypothalamus and pituitary gland via muscarinic and nicotinic receptors, thereby altering thyroid function (12).

Phenoxybenzoic acid (3P) and 4-fluoro-3-phenoxybenzoic acid (4F3PB) are metabolites of pyrethroids, a class of broad-spectrum insecticides popularly used in agriculture and indoor pest control. These compounds cause paralysis and death of the insect pests by interfering with their nervous system excitability (4). These pesticides are ubiquitously used for controlling mosquitoes, moths, fleas, and other pests in fields, greenhouses, homes, and public places. Although this pesticide type has substantial insecticidal activity and is less toxic to humans and mammals, its adverse health effects cannot be ignored. The pyrethroid metabolites have been found to act as thyroid disruptors, affecting the hypothalamic–pituitary-thyroid axis to varying degrees (13). *In vitro* research has shown that pyrethroids can antagonize thyroid receptors and consequently block the thyroid axis, with the possibility of pyrethroids or their metabolites interacting with androgens or estrogen receptors also being indicated (14). Furthermore, *in vivo* experiments have examined the effects of two pyrethroids (permethrin and beta-cypermethrin) and three pyrethroid metabolites (3-phenoxybenzyl alcohol, 3-phenoxybenzaldehyde, and 3-phenoxybenzoic acid) in zebrafish models. The results suggested that pyrethroid insecticides and their metabolites influenced thyroid signaling, motor behavior, and embryonic development in the zebrafish, implying that thyroid disruption may be involved in abnormal larva development (15).

The human body absorbs pesticides not only during agricultural work but also via fruit and vegetable dietary intake as well as direct pesticide consumption by those attempting suicide. However, pesticide absorption in these population groups is generally linked to two or more pesticides and not a single pesticide. Hence, the effect of multiple pesticides on thyroid function also requires attention. Despite this understanding, most current research focuses on the impact of individual pesticides on the human thyroid function index. Therefore, this study investigated the combined effect of OP, pyrethroid, and herbicide metabolites on thyroid function.

## 2 Materials and methods

### 2.1 Analysis of clinical patient and control data

#### 2.1.1 Patients and controls

This retrospective study selected 60 patients hospitalized for pyrethroid pesticide poisoning and 60 healthy control participants who had undergone health examinations at Binhai County People’s Hospital between June 2022 and June 2023. This study complied with the criteria outlined in the Declaration of Helsinki (as revised in 2013) and was approved by the Ethics Committee of Binhai County People’s Hospital (approval no: 2023-BHKYLL-018). All patients and controls or their relatives provided signed informed consent before study enrollment.

The patient inclusion criteria were as follows: (1) pyrethroid pesticide exposure and (2) age > 20 years. Patients were excluded if they met the following exclusion criteria: (1) previous thyroid disease history or (2) pregnancy or lactation. A total of 60 patients were enrolled based on these criteria.

#### 2.1.2 Data collection

All patient data within 48 h after admission were reviewed and collected from our hospital’s electronic medical records as raw data. Acquired data included age, sex, marriage, education, smoking status, alcohol consumption, and laboratory thyroid test results (FT3, FT4, TSH, TT3, TT4, TgAb, and TPOAb levels). Details of the thyroid function indicators are shown in Supplementary Table S1.

### 2.2 Analysis of participants in the NHANES database

#### 2.2.1 Study design and population

NHANES is a series of cross-sectional, nationally representative surveys conducted annually by the National Center for Health Statistics of the Centers for Disease Control and Prevention to estimate and assess the health and nutritional statuses as well as the potential risk factors of the non-institutionalized civilian population in the United States. All included participants have provided written informed consent before survey inclusion. All NHANES studies receive approval from the National Health Statistics Research Ethics Review Board.^1^ All programs comply with the relevant guidelines and regulations.^2^

**Figure 1:**
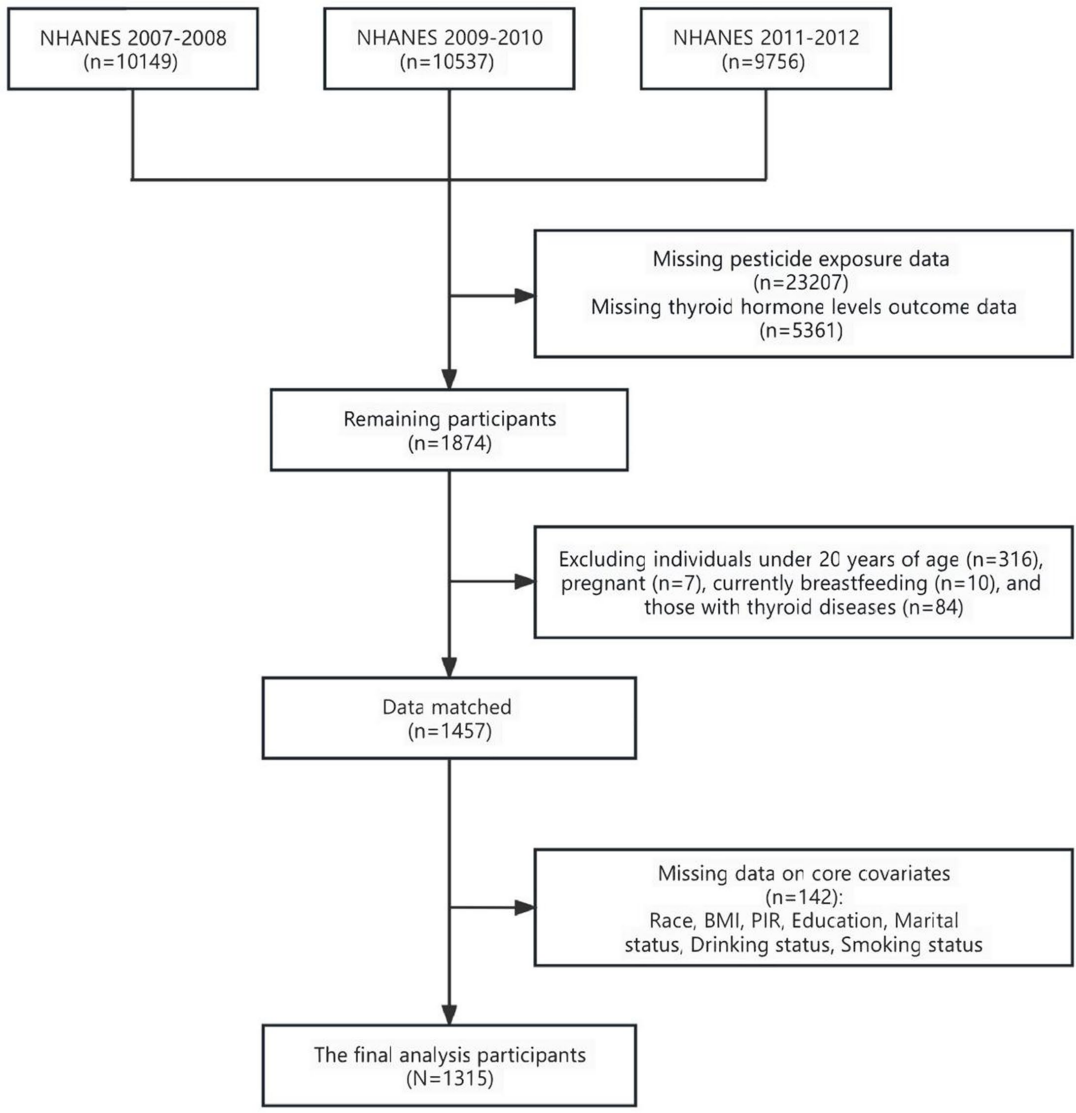
Flowchart of study participant selection in the NHANES 2007–2012.

Three open-access consecutive surveys were retrieved from the NHANES website: 2007–2008, 2009–2010, and 2011–2012. The collected data included demographic, inspection, laboratory, and questionnaire information. The total sample size in the included surveys was 30,442 individuals. Participants <20 years of age and those lacking data on the levels of pesticide metabolites, thyroid hormones, and antibodies were excluded (*n* = 28,884). Additionally, pregnant and lactating women and those with pre-existing thyroid conditions were excluded (*n* = 101). Lastly, individuals with missing covariate data, such as education, marriage, and smoking status, were excluded (*n* = 142). Ultimately, 1,315 individuals were included (Figure 1).

#### 2.2.2 Measurement of pesticide metabolites

The body primarily eliminates absorbed pesticides by excreting their associated metabolites via urine. The metabolites of OPs mainly comprise 2,4-D. Furthermore, 4F3PB, TDDC, and 3P are pyrethroid metabolites, while PN is primarily a herbicide metabolite. Therefore, an urine concentration test for the above metabolites is crucial to detect pesticide poisoning and determine the disease status in patients with pesticide poisoning.

The levels of various pesticide metabolites in the urine were measured and quantified from the participants’ urine matrix using an automated solid-phase extraction system. The samples were analyzed via HPLC and a triple quadrupole mass spectrometer with a heated electrospray ionization source (16). The lower limit concentration of 2,4-D detection was 0.15 μg/L; 4F3PB, 0.10 μg/L; 3P, 0.10 μg/L; PN, 0.10 μg/L; and TDDC, 0.60 μg/L. Furthermore, data below the lower limit of their detection (LOD) were specified as LOD divided by the square root of 2 to improve the statistical power and accuracy of effect estimation (17). The official website (NHANES 2007–2012) provides additional laboratory information concerning the applied methods and procedures.

#### 2.2.3 Thyroid hormone

Thyroid blood specimens were processed, stored, and shipped to Collaborative Laboratory Services, Ottumwa, Iowa, United States. A competitive binding immunoenzymatic assay was used to detect TT3, TT4, and FT3 levels. Additionally, a two-step enzyme immunoassay was used to determine FT4 concentration, while a third-generation two-site immunoenzymatic (“sandwich”) assay was employed to estimate TSH levels. Lastly, a simultaneous one-step “sandwich” assay was applied to obtain Tg levels, whereas a continuous two-step immunoenzymatic “sandwich” assay was performed to measure TgAb and TPOAb concentrations. The specific details of the methods are available in NHANES 2007–2012.

#### 2.2.4 Covariates

Based on previous studies, relevant variables were selected as the predictors of the preliminary analysis to control for potential confounding effects. The selected covariables included age, sex, race, education level, and obesity. The statistical model was then adjusted using the covariates to reduce the confounding bias on the research results. The adjusted covariates included demographic characteristics such as (a) age (20–39, 40–59, and ≥ 60 years), (b) race (Mexican Americans, non-Hispanic whites, non-Hispanic blacks, other Hispanics, and other races including multiracial groups), (c) gender (male and female), (d) marital status (married, unmarried, and other status including divorced or widowed), and (e) education (below high school, high school, and above high school), as well as characteristics such as (f) smoking status (never smoker, former smoker, and current smoker), (g) alcohol consumption (non-drinkers and moderate drinkers: 1–2 drinks/day for men and 1 drink/day for women; and heavy drinkers: >2 drinks/day for men and > 1 drink/day for women), and (h) body mass index (BMI, normal: 25 kg/m^2^, overweight: 25–30 kg/m^2^, and obese: ≥30 kg/m^2^). The uniform interviews and questionnaires were completed by trained medical professionals.

### 2.3 Statistical analysis

All statistical analyses were performed using StataMP 17 version, GraphPad Prism 9.5, and R 4.2.3 software, with three statistical packages (“rms,” “gWQS,” and “bkmr”). A *p*-value of <0.05 (bilateral) was considered statistically significant.

#### 2.3.1 Descriptive statistical analysis

Normal continuous data were expressed as mean ± standard deviation (mean ± SD), whereas skewed continuous data were presented as median and interquartile distance (IQR). Furthermore, categorical variables were denoted as numbers (percentages). The differences between the age groups were assessed using an independent sample *T*-test and Mann–Whitney U test for continuous variables and the chi-square test for categorical variables. The pesticide metabolite levels and thyroid function results were naturally log-transformed in an approximately normal distribution. Spearman’s rank coefficient was applied to measure the correlation between each pesticide metabolite.

#### 2.3.2 Bayesian kernel machine regression model

The potential complex nonlinear or linear relationships between pesticide metabolites and the various thyroid hormones were evaluated using the BKMR model. BKMR is a nonparametric model combining Bayesian and statistical learning techniques, providing strong adaptability and good model-fitting ability for highly correlated variables common in the environmental epidemiological community (18, 19). Further, we modeled the exposure-response function using a Gaussian approach, followed by the application of the Markov chain Monte Carlo algorithm for 25,000 iterations. Thus, the overall effects between pesticide metabolites and different thyroid hormones were visualized using BKMR.

#### 2.3.3 Linear regression model

A weighted LRM was employed to determine the correlation between the pesticide metabolites and thyroid function indicators. Model 1 was not adjusted for confounding factors, while model 2 was adjusted for age, sex, race/ethnicity, education level, smoking status, alcohol consumption, and BMI. Additionally, age group analysis was used to understand the effects of pesticide metabolites on thyroid function indexes across different ages. Moreover, we used WTMEC2YR to provide weights for all data to ensure nationally representative results. Finally, we conducted several restricted cubic spline (RCS) analyses to explore the nonlinear dose relationship between pesticide metabolite exposure in the entire population and thyroid hormones.

#### 2.3.4 Weighted quantile sum model

Finally, a WQS regression method was utilized to investigate the effect of the pesticide mixture on thyroid function indicators. The WQS regression technique combines different highly correlated compounds into a composite index, followed by regression analysis on that index. Additionally, WQS regression uses the bootstrap method to assign individual weights to each metabolite, allowing the identification of relatively critical components in the pesticide mixture (20). The weight of each metabolite ranges from 0 to 1, and the sum of the weights is 1. The effect of the metabolite on thyroid function increases with the increase in its weight value. The WQS approach provides better coverage of real-life mixed exposures and is more sensitive than univariate analysis in identifying vital predictors. In this study, 40% of the random samples were used to test and 60% to verify the data.

## 3 Results

### 3.1 Characteristics of the clinical study patients and controls

**Figure 2:**
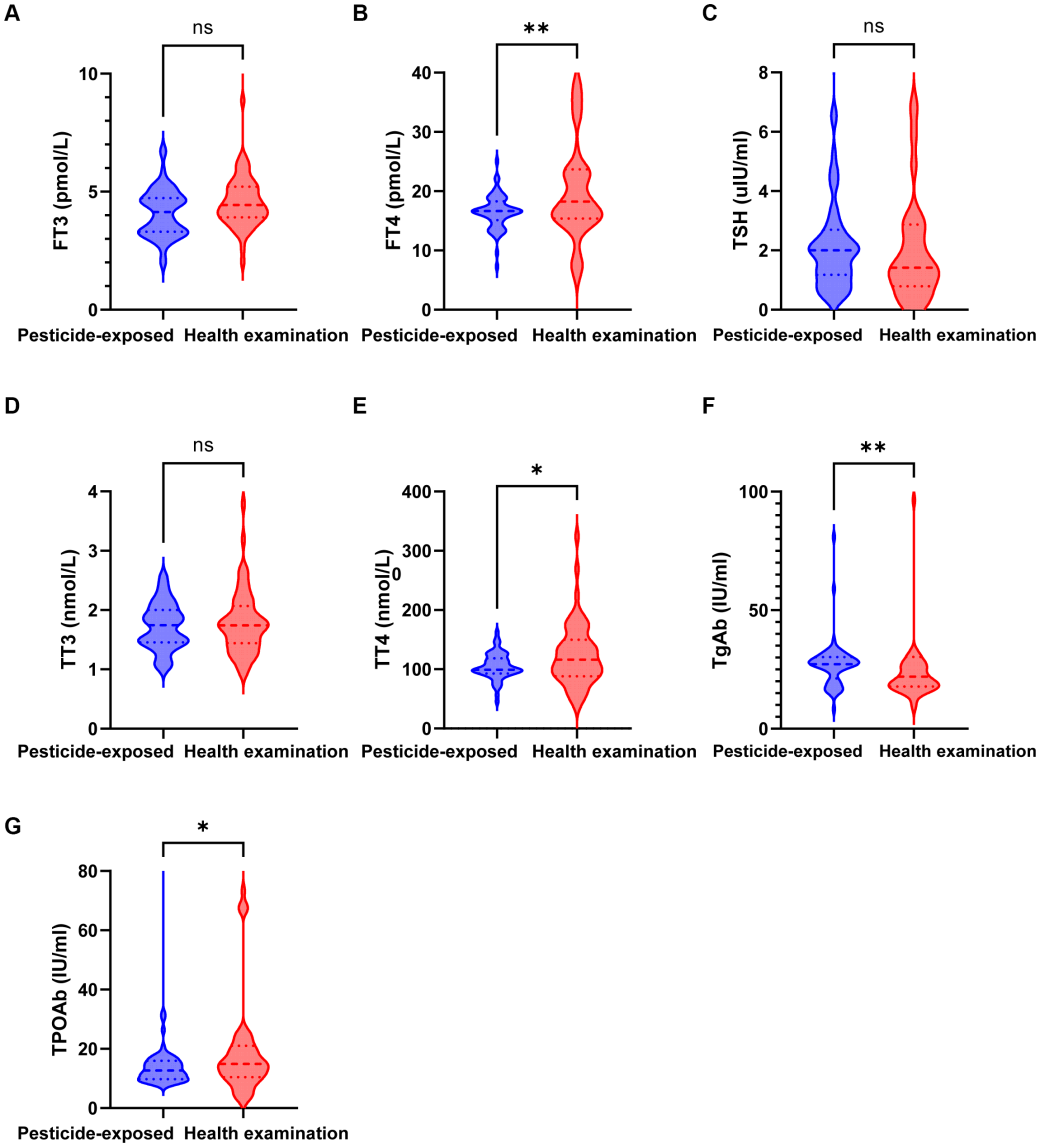
Expression levels of thyroid function indicators in the pesticide-exposed and health examination control groups. **(A)** The level of FT3 between the two groups; **(B)** The level of FT4 between the two groups; **(C)** The level of TSH between the two groups; **(D)** The level of TT3 between the two groups; **(E)** The level of TT4 between the two groups; **(F)** The level of TgAb between the two groups; **(G)** The level of TPOAb between the two groups.

A total of 120 patients and controls from our hospital were included, comprising 60 hospitalized patients with pyrethroid exposure and 60 healthy control participants who underwent physical examination. As depicted in Table 1 and Figure 2, the mean ± SD of the levels of the thyroid function indicators FT4 (22.304 ± 16.188 pmol/L), TT4 (123.73 ± 52.232 nmol/L), TgAb (68.185 ± 119.849 IU/mL), and TPOAb (32.041 ± 60.602 IU/mL) in the healthy group significantly differed from those in the pesticide-exposed group (FT4: 16.488 ± 2.877 pmol/L, *p* = 0.008; TT4: 104.834 ± 21.865 nmol/L, *p* = 0.012; 27.214 ± 10.094 IU/mL, *p* = 0.011; and TPOAb: 14.699 ± 10.316 IU/mL, *p* = 0.033). Sex, age, education, marital status, smoking status, and alcohol consumption were not statistically significant.

**Table 1:** Baseline characteristics of the clinical patients and controls.

Variables	Total (*n* = 120)	Health examination control group (*n* = 60)	Pesticide-exposed patient group (*n* = 60)	*p*-value
Gender, *n* (%)				0.449
Male	44 (36.667)	24 (40)	20 (33.333)	
Female	76 (63.333)	36 (60)	40 (66.667)	
Age, *n* (%)				0.063
20–39 years	29 (24.167)	20 (33.333)	9 (15)	
40–59 years	38 (31.667)	17 (28.333)	21 (35)	
≥60 years	53 (44.167)	23 (38.333)	30 (50)	
Educational level (%)				0.929
Less than high school	74 (61.667)	38 (63.333)	36 (60)	
High school graduate	17 (14.167)	8 (13.333)	9 (15)	
College or above	29 (24.167)	14 (23.333)	15 (25)	
Marital status (%)				0.81
Never married	21 (17.5)	11 (18.333)	10 (16.667)	
Married	99 (82.5)	49 (81.667)	50 (83.333)	
Smoking status, *n* (%)				0.338
No	92 (76.667)	44 (73.333)	48 (80)	
Yes	28 (23.333)	16 (26.667)	12 (20)	
Drinking status, *n* (%)				0.071
No	85 (70.833)	38 (63.333)	47 (78.333)	
Yes	35 (29.167)	22 (36.667)	13 (21.667)	
Thyroid function index				
FT3 (pmol/L)	4.748 ± 4.775	5.397 ± 6.643	4.098 ± 1.004	0.139
FT4 (pmol/L)	19.396 ± 11.94	22.304 ± 16.188	16.488 ± 2.877	**0.008**
TSH (uIU/mL)	2.412 ± 2.702	2.122 ± 2.114	2.703 ± 3.175	0.24
TT3 (nmol/L)	1.809 ± 0.76	1.895 ± 1	1.724 ± 0.388	0.22
TT4 (nmol/L)	114.282 ± 40.984	123.73 ± 52.232	104.834 ± 21.865	**0.012**
TgAb (IU/mL)	47.7 ± 87.151	68.185 ± 119.849	27.214 ± 10.094	**0.011**
TPOAb (IU/mL)	23.37 ± 44.152	32.041 ± 60.602	14.699 ± 10.316	**0.033**

Continuous data are displayed as mean ± standard deviation. Categorical variables are presented as *n* (%), where *n* is the number of patients or controls and % is the weighted percentage. *p* < 0.05 was considered statistically significant. Not significant (ns), *p* > 0.05; *, *p* < 0.05; **, *p* < 0.01. The bold type means statistically significant.

### 3.2 Relationship between pesticide poisoning and thyroid function indicators

Linear regression modeling was used to evaluate the correlation between pesticide poisoning and thyroid function indicators and to understand whether any changes occurred in these parameters after pesticide poisoning (Table 2). The results of model 1, which was not adjusted for covariates, showed that pesticide poisoning in patients was negatively correlated with FT4 (*p* = 0.007), TT4 (*p* = 0.011), TgAb (*p* = 0.009), and TPOAb (*p* = 0.031). Further, considering that numerous potential factors can affect the thyroid function indicators of the patients, covariates such as age and gender were added to re-establish an LRM (model 2) to evaluate the correlation between them (Table 2). The analysis demonstrated that pesticide poisoning in patients was negatively correlated with FT4 (*p* = 0.007), TT4 (*p* = 0.01), TgAb (*p* = 0.008), and TPOAb (*p* = 0.04). Thus, pesticide poisoning was negatively correlated with the thyroid function indicators FT4, TT4, TgAb, and TPOAb, even after adjusting for relevant confounders.

**Table 2:** Linear regression analysis of the relationship between pesticide exposure in clinical patients and thyroid function indicators.

Outcome	Model 1		Model 2	
	β (95% CI)	*p*-value	β (95% CI)	*p*-value
**FT3**	−1.299 (−3.016 to 0.419)	0.137	−1.224 (−3.055 to 0.607)	0.188
FT4	**−5.816 (−10.019 to − 1.613)**	**0.007**	**−6.192 (−10.667 to − 1.718)**	**0.007**
TSH	0.582 (−0.393 to 1.557)	0.24	0.549 (−0.447 to 1.546)	0.277
TT3	−0.171 (−0.4451 to 0.103)	0.219	−0.147 (−0.432 to 0.137)	0.306
TT4	**−18.896 (−33.372 to − 4.420)**	**0.011**	**−20.163 (−35.437 to − 4.889)**	**0.01**
TgAb	**−40.971 (−71.719 to − 10.222)**	**0.009**	**−42.868 (−74.425 to − 11.311)**	**0.008**
TPOAb	**−17.342 (−33.058 to − 1.626)**	**0.031**	**−16.854 (−32.946 to − 0.763)**	**0.04**

Model 1: Non-adjusted model.

Model 2: Adjusted for gender, age, education, marital status, alcohol use, and smoking status.

95% CI, 95% confidence interval. Bold values indicate statistical significance at *p* < 0.05.

### 3.3 Association between the metabolic mixture of pesticides and thyroid function indicators (based on the BKMR model)

**Figure 3:**
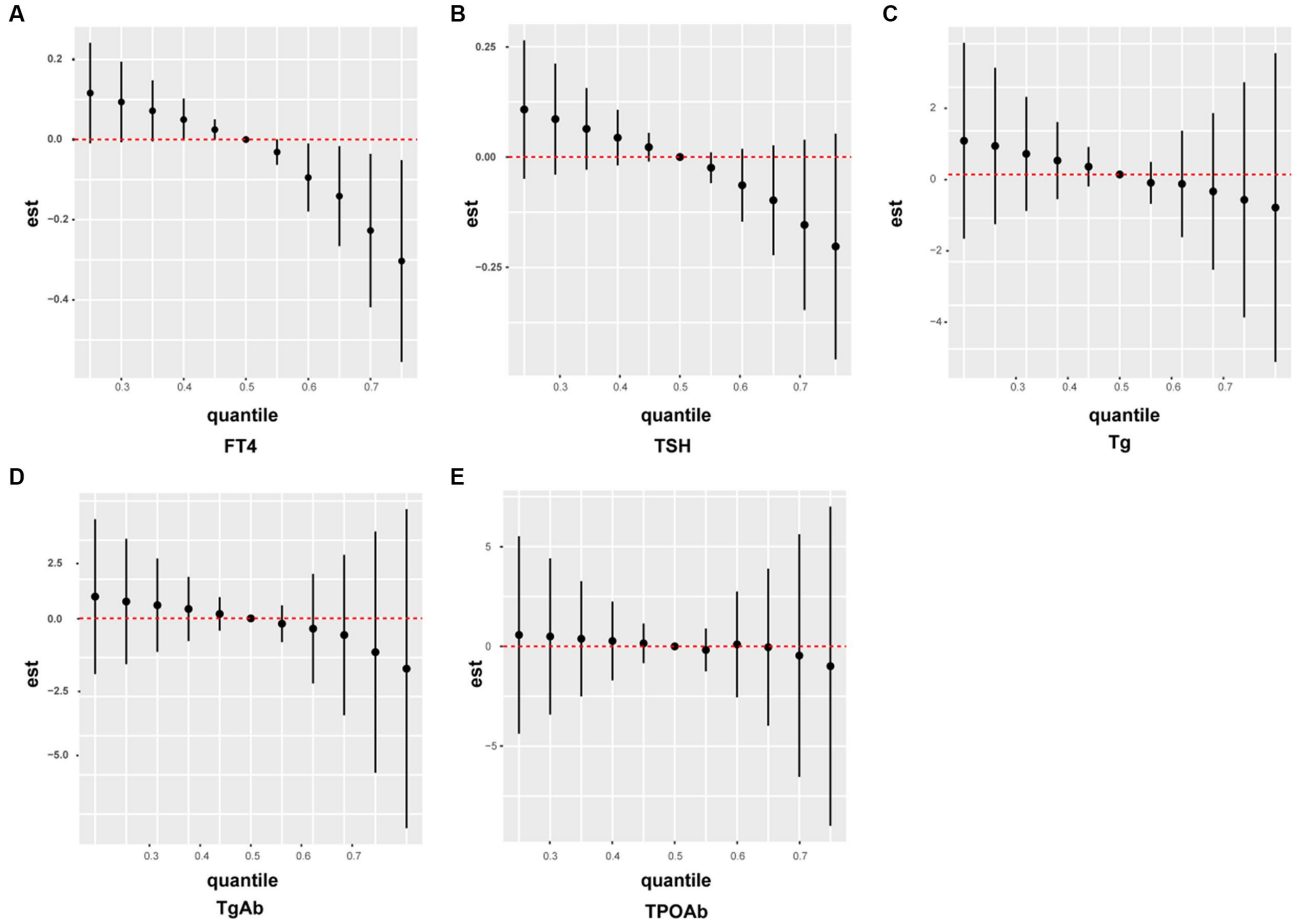
A BKMR model to estimate the combined risk effect of the pesticide mixture on thyroid function indicators in the general population. The models were adjusted for gender, age, race, education, marital status, body mass index, smoking status, and alcohol consumption. **(A)** FT4; **(B)** TSH; **(C)** Tg; **(C)** TgAb; **(E)** TPOAb.

We developed a BKMR model based on the NHANES data, to understand the effect of the overall metabolic mixture of pesticides on the indicators of thyroid function. Figure 3 illustrates the effects of the mixture of the five pesticide metabolites 2,4-D, 4F3PB, 3P, PN, and TDDC on thyroid function indexes. The mixture exhibited a negative correlation with FT4, TSH, and Tg and with TgAb and TPOAb after the 60th and 70th percentiles, respectively. All these results were consistent with our clinical data, indicating that the metabolic mixture of the pesticides was negatively correlated with thyroid function indexes.

### 3.4 Basic participant information in the NHANES 2007–2012

Among the 30,442 participants in the NHANES 2007–2012, 1,315 were eventually included according to our study inclusion and exclusion criteria. Table 3 displays the basic characteristic information of the study participants grouped by age. The age groups of 20–39, 40–59, and ≥ 60 years comprised 437, 430, and 448 individuals, respectively. The mean ± SD of the 3P levels was significantly different across the age groups of 20–39 (1.528 ± 4.145 μg/L), 40–59 (1.617 ± 3.802 μg/L), and ≥ 60 (0.954 ± 2.256 μg/L) years. Similarly, the mean ± SD of the levels of FT3 (20–39 years: 3.341 ± 0.383 pg./mL, 40–59 years: 3.183 ± 0.415 pg./mL, and ≥ 60 years: 2.989 ± 0.35 pg./mL), FT4 (20–39 years: 0.78 ± 0.127 ng/dL, 40–59 years: 0.775 ± 0.165 ng/dL, and ≥ 60 years: 0.813 ± 0.176 ng/dL), TSH (20–39 years: 1.758 ± 1.263 mIU/L, 40–59 years: 2.03 ± 3.11 mIU/L, and ≥ 60 years: 2.445 ± 3.328 mIU/L), TT3 (20–39 years: 118.043 ± 20.982 ng/dL, 40–59 years: 114.286 ± 24.429 ng/dL, and ≥ 60 years: 103.089 ± 20.938 ng/dL), and TgAb (20–39 years: 10.722 ± 9.299 IU/mL, 40–59 years: 8.819 ± 81.876 IU/mL, and ≥ 60 years: 19.664 ± 114.705 IU/mL) were significantly different, with all *p*-values <0.05. Race, marital status, smoking status, alcohol consumption, and BMI were also significantly different between all age groups, with all *p*-values <0.05.

**Table 3:** Weighted characteristics of the NHANES participants from 2007 to 2012 according to age groups.

Variables	Total (*n* = 1,315)	20–39 years (*n* = 437)	40–59 years (*n* = 430)	≥60 years (*n* = 448)	*p*-value
Gender, *n* (%)					0.271
Male	652 (49.582)	217 (49.657)	225 (52.326)	210 (46.875)	
Female	663 (50.418)	220 (50.343)	205 (47.674)	238 (53.125)	
Race, *n* (%)					**0.003**
Mexican American	232 (17.643)	92 (21.053)	81 (18.837)	59 (13.17)	
Other Hispanic	145 (11.027)	45 (10.297)	55 (12.791)	45 (10.045)	
Non-Hispanic White	630 (47.909)	187 (42.792)	192 (44.651)	251 (56.027)	
Non-Hispanic Black	245 (18.631)	87 (19.908)	84 (19.535)	74 (16.518)	
Other races	63 (4.791)	26 (5.95)	18 (4.186)	19 (4.241)	
Educational level (%)					0.337
Below high school	371 (28.213)	121 (27.689)	113 (26.279)	137 (30.58)	
High school	317 (24.106)	99 (22.654)	103 (23.953)	115 (25.67)	
Above high school	627 (47.681)	217 (49.657)	214 (49.767)	196 (43.75)	
Marital status (%)					**<0.001**
Married	236 (17.947)	176 (40.275)	43 (10)	17 (3.795)	
Never married	768 (58.403)	225 (51.487)	282 (65.581)	261 (58.259)	
Widowed/Divorced/Separated	311 (23.65)	36 (8.238)	105 (24.419)	170 (37.946)	
Smoking status (%)					**<0.001**
Never	685 (52.091)	251 (57.437)	207 (48.14)	227 (50.67)	
Former	317 (24.106)	56 (12.815)	96 (22.326)	165 (36.83)	
Current	313 (23.802)	130 (29.748)	127 (29.535)	56 (12.5)	
Alcohol use (%)					**<0.001**
Non-drinker	221 (16.806)	55 (12.586)	58 (13.488)	108 (24.107)	
Moderate drinker	485 (36.882)	115 (26.316)	153 (35.581)	217 (48.438)	
Heavy drinker	609 (46.312)	267 (61.098)	219 (50.93)	123 (27.455)	
Body mass index (%)					**<0.001**
<25 kg/m^2^	381 (28.973)	161 (36.842)	103 (23.953)	117 (26.116)	
25–29.9 kg/m^2^	472 (35.894)	147 (33.638)	162 (37.674)	163 (36.384)	
≥30 kg/m^2^	462 (35.133)	129 (29.519)	165 (38.372)	168 (37.5)	
Pesticide metabolic compound					
2,4-D (μg/L)	0.595 ± 1.424	0.528 ± 1.04	0.641 ± 2.008	0.616 ± 1.016	0.465
4F3PB (μg/L)	0.088 ± 0.122	0.087 ± 0.097	0.086 ± 0.095	0.092 ± 0.159	0.746
3P (μg/L)	1.361 ± 3.498	1.528 ± 4.145	1.617 ± 3.802	0.954 ± 2.256	**0.009**
PN (μg/L)	1.366 ± 2.736	1.444 ± 2.964	1.268 ± 2.325	1.386 ± 2.868	0.627
TDDC (μg/L)	1.257 ± 3.354	1.154 ± 3.119	1.484 ± 3.728	1.14 ± 3.189	0.233
Thyroid function index					
FT3 (pg/mL)	3.169 ± 0.409	3.341 ± 0.383	3.183 ± 0.415	2.989 ± 0.35	**<0.001**
FT4 (ng/dL)	0.79 ± 0.158	0.78 ± 0.127	0.775 ± 0.165	0.813 ± 0.176	**<0.001**
TSH (mIU/L)	2.081 ± 2.745	1.758 ± 1.263	2.03 ± 3.11	2.445 ± 3.328	**<0.001**
TT3 (ng/dL)	111.72 ± 23.042	118.043 ± 20.982	114.286 ± 24.429	103.089 ± 20.938	**<0.001**
TT4 (μg/dL)	7.862 ± 1.672	7.803 ± 1.656	7.764 ± 1.676	8.014 ± 1.677	0.057
Tg (ng/mL)	16.333 ± 35.266	15.315 ± 31.046	14.103 ± 19.414	19.467 ± 48.357	0.06
TgAb (IU/mL)	10.155 ± 82.148	10.722 ± 9.299	8.819 ± 81.876	19.664 ± 114.705	**0.005**
TPOAb (IU/mL)	19 ± 87.976	14.167 ± 79.862	25.638 ± 108.061	17.345 ± 72.499	0.14

Continuous data are displayed as mean ± standard deviation. Categorical variables are presented as *n* (%), where *n* is the number of participants and % is the weighted percentage. *p* < 0.05 was considered statistically significant. The bold type means statistically significant.

Next, we conducted a Spearman correlation analysis to understand the relationship between the metabolites of the different pesticides. Supplementary Figure S1 and Supplementary Table S2 present the correlation coefficients (*r*) between various metabolites, ranging from 0.033 to 0.504. In particular, the *r* above 0.5 is 3P and TDDC, which are moderately correlated. Furthermore, 3P and PN exhibited a slight correlation (*r* = 0.5), while the remaining metabolites had a weak correlation (*r* < 0.3). Lastly, Pearson’s correlation coefficients between all metabolites had *p*-values of <0.05, except for that between 2,4-D and TDDC.

### 3.5 Association between pesticide metabolites and thyroid function indicators (based on the linear regression model)

Table 4 shows the association between each pesticide metabolite and thyroid function indicators in the LRM. In model 1 (without adjustment for any confounding factors), 2,4-D and FT3 (*p* = 0.041) as well as 4F3PB and FT4 (*p* = 0.003) were positively correlated. Additionally, a negative association was detected between 4F3PB and Tg (*p* = 0.001), 4F3PB and TgAb (*p* = 0.006), 3P and TgAb (*p* = 0.006), 3P and TPOAb (*p* = 0.03), PN and TSH (*p* = 0.003), PN and TT4 (*p* = 0.031), and TDDC and TPOAb (*p* < 0.001). After correcting for the confounding factors such as age, sex, race, education, marital status, smoking status, and alcohol consumption in model 2, the correlations between 2,4-D and FT3 (*p* = 0.039) as well as 4F3PB and FT4 (*p* = 0.005) remained. Moreover, the negative correlations persisted between 4F3PB and Tg (*p* = 0.027), 4F3PB and TgAb (*p* = 0.024), 3P and TgAb (*p* = 0.041), 3P and TPOAb (*p* = 0.047), PN and FT4 (*p* = 0.034), PN and TSH (*p* = 0.011), PN and TT4 (*p* = 0.049), and TDDC and TPOAb (*p* = 0.003).

**Table 4:** Weighted linear regression analysis of the relationship between pesticide exposure and thyroid function indexes in the NHANES participants from 2007 to 2012.

Exposure outcome		Model 1		Model 2	
		β (95% CI)	*p*-value	β (95% CI)	*p*-value
2,4-D					
	FT3	**0.017 (0.00–10.033)**	**0.041**	**0.019 (0.001–0.038)**	**0.039**
	FT4	0.002 (−0.005 to 0.010)	0.536	0.002 (−0.005 to 0.009)	0.525
	TSH	0.109 (−0.166 to 0.383)	0.438	0.109 (−0.162 to 0.380)	0.43
	TT3	0.431 (−0.408 to 1.270)	0.313	0.457 (−0.354 to 1.267)	0.269
	TT4	−0.029 (−0.118 to 0.061)	0.53	−0.006 (−0.088 to 0.076)	0.892
	Tg	−0.081 (−0.834 to 0.672)	0.833	0.210 (−0.546 to 0.966)	0.586
	TgAb	0.621 (−0.998 to 2.240)	0.452	0.488 (−1.137 to 2.113)	0.556
	TPOAb	1.141 (−2.272 to 4.554)	0.512	2.012 (−1.361 to 5.384)	0.242
4F3PB					
	FT3	−0.142 (−0.361 to 0.078)	0.205	−0.108 (−0.289 to 0.0742)	0.246
	FT4	**0.112 (0.038–0.187)**	**0.003**	**0.107 (0.033–0.182)**	**0.005**
	TSH	−0.077 (−1.054 to 0.900)	0.878	−0.052 (−1.012 to 0.907)	0.914
	TT3	−8.388 (−17.922 to 1.147)	0.085	−6.320 (−14.120 to 1.481)	0.112
	TT4	0.699 (−0.046 to 1.443)	0.066	0.718 (−0.019 to 1.455)	0.056
	Tg	**−9.226 (−14.84 to −3.611)**	**0.001**	**−7.173 (−13.532 to −0.813)**	**0.027**
	TgAb	**−7.835 (−13.383 to −2.287)**	**0.006**	**−8.765 (−16.399 to −1.130)**	**0.024**
	TPOAb	−9.749 (−37.648 to 18.149)	0.493	−10.683 (−36.069 to 14.704)	0.409
3P					
	FT3	0.002 (−0.005 to 0.009)	0.49	−0.001 (−0.007 to 0.005)	0.795
	FT4	0.001 (−0.001 to 0.004)	0.331	0.002 (−0.001 to 0.004)	0.192
	TSH	−0.020 (−0.0407 to 0.001)	0.059	−0.017 (−0.039 to 0.004)	0.111
	TT3	−0.081 (−0.517 to 0.354)	0.714	−0.186 (−0.595 to 0.223)	0.373
	TT4	−0.003 (−0.035 to 0.030)	0.868	0.003 (−0.023 to 0.029)	0.804
	Tg	0.577 (−0.343 to 1.498)	0.219	0.618 (−0.278 to 1.515)	0.176
	TgAb	**−0.368 (−0.632 to − 0.104)**	**0.006**	**−0.282 (−0.553 to − 0.011)**	**0.041**
	TPOAb	**−0.783 (−1.492 to − 0.075)**	**0.03**	**−0.795 (−1.579 to − 0.011)**	**0.047**
PN					
	FT3	−0.005 (−0.018 to 0.009)	0.488	−0.006 (−0.018 to 0.007)	0.38
	FT4	−0.002 (−0.005 to 0.001)	0.099	−0.003 (−0.005 to − 0.001)	0.034
	TSH	**−0.044 (−0.073 to − 0.016)**	**0.003**	**−0.035 (−0.061 to − 0.008)**	**0.011**
	TT3	−0.387 (−0.883 to 0.108)	0.126	−0.270 (−0.739 to 0.199)	0.259
	TT4	**−0.042 (−0.080 to − 0.004)**	**0.031**	**−0.042 (−0.086 to − 0.001)**	**0.049**
	Tg	0.299 (−0.313 to 0.913)	0.337	0.329 (−0.273 to 0.932)	0.284
	TgAb	1.085 (−1.327 to 3.498)	0.378	1.076 (−1.400 to 3.552)	0.394
	TPOAb	−0.715 (−1.748 to 0.317)	0.174	−0.302 (−1.458 to 0.854)	0.609
TDDC					
	FT3	0.001 (−0.006 to 0.007)	0.801	0.001 (−0.004 to 0.006)	0.732
	FT4	0.001 (−0.001 to 0.004)	0.278	0.001 (−0.001 to 0.003)	0.339
	TSH	−0.020 (−0.049 to 0.008)	0.154	−0.022 (−0.052 to 0.008)	0.15
	TT3	−0.127 (−0.519 to 0.265)	0.525	−0.079 (−0.474 to 0.317)	0.696
	TT4	0.001 (−0.026 to 0.027)	0.971	0.001 (−0.022 to 0.023)	0.95
	Tg	0.770 (−0.603 to 2.143)	0.271	0.748 (−0.607 to 2.104)	0.279
	TgAb	−0.151 (−0.886 to 0.584)	0.686	−0.117 (−0.900 to 0.665)	0.769
	TPOAb	**−1.370 (−2.080 to − 0.660)**	**<0.001**	**−1.228 (−2.037 to − 0.420)**	**0.003**

Model 1: Non-adjusted model.

Model 2: Gender, age, race, education, marital status, body mass index, alcohol use, and smoking status.

95% CI, 95% confidence interval. Bold values indicate statistical significance at *p* < 0.05.

Next, we re-established an LRM to assess the relationship between exposure to pesticide metabolites and thyroid function indicators across different age groups (Supplementary Tables S3–S7). Our results showed that 4F3PB was negatively correlated with Tg (*p* < 0.001) and TgAb (*p* = 0.046), while PN demonstrated a negative correlation with TSH (*p* = 0.001) and TPOAb (*p* = 0.046) in the 20–39 years age group. In the 40–59 years age group, 4F3PB showed a positive correlation with FT4 (*p* = 0.024) and negative correlations with TSH (*p* = 0.001) and TPOAb (*p* = 0.005). Furthermore, 3P was negatively associated with TSH (*p* = 0.014), TgAb (*p* = 0.043), and TPOAb (*p* < 0.001), whereas PN exhibited a negative correlation with TSH (*p* = 0.017) and TDDC was negatively correlated with TSH (*p* = 0.001) and TPOAb (*p* = 0.001). In the ≥60 years age group, a positive correlation was found between 2,4-D and FT3 (*p* = 0.039), while 4F3PB was positively correlated with FT4 (*p* < 0.001) and negatively correlated with FT3 (*p* < 0.001), TT3 (*p* < 0.001), TT4 (*p* < 0.001), Tg (*p* < 0.001), TgAb (*p* = 0.004), and TPOAb (*p* < 0.001). Additionally, whereas negative correlations were demonstrated between PN and TT3 (*p* = 0.033) and TDDC and TPOAb (*p* = 0.034).

### 3.6 Relationship between pesticide metabolites and thyroid function index (according to the restricted curve spline model)

An RCS model was employed to estimate the dose–response relationship between individual pesticide metabolites and thyroid function indicators (Supplementary Figures S2–S6). In this analysis, 2,4-D was positively correlated with FT3 (Supplementary Figure S2), while 4F3PB exhibited positive correlations with FT4 and TT4 and negative correlations with TT3, Tg, and TPOAb (Supplementary Figure S3). Furthermore, 3P showed positive associations with FT3, FT4, and Tg and negative correlations with TgAb and TPOAb (Supplementary Figure S4), whereas PN demonstrated a negative correlation with TSH and positive correlations with Tg and TgAb (Supplementary Figure S5). Finally, TDDC at <8.731 μg/L was positively correlated with FT4 and Tg and negatively correlated with TSH, whereas TDDC at >8.731 μg/L was positively correlated with TSH (Supplementary Figure S6).

### 3.7 Relationship between pesticide metabolites and thyroid function index (based on the weighted quantile sum model)

**Figure 4:**
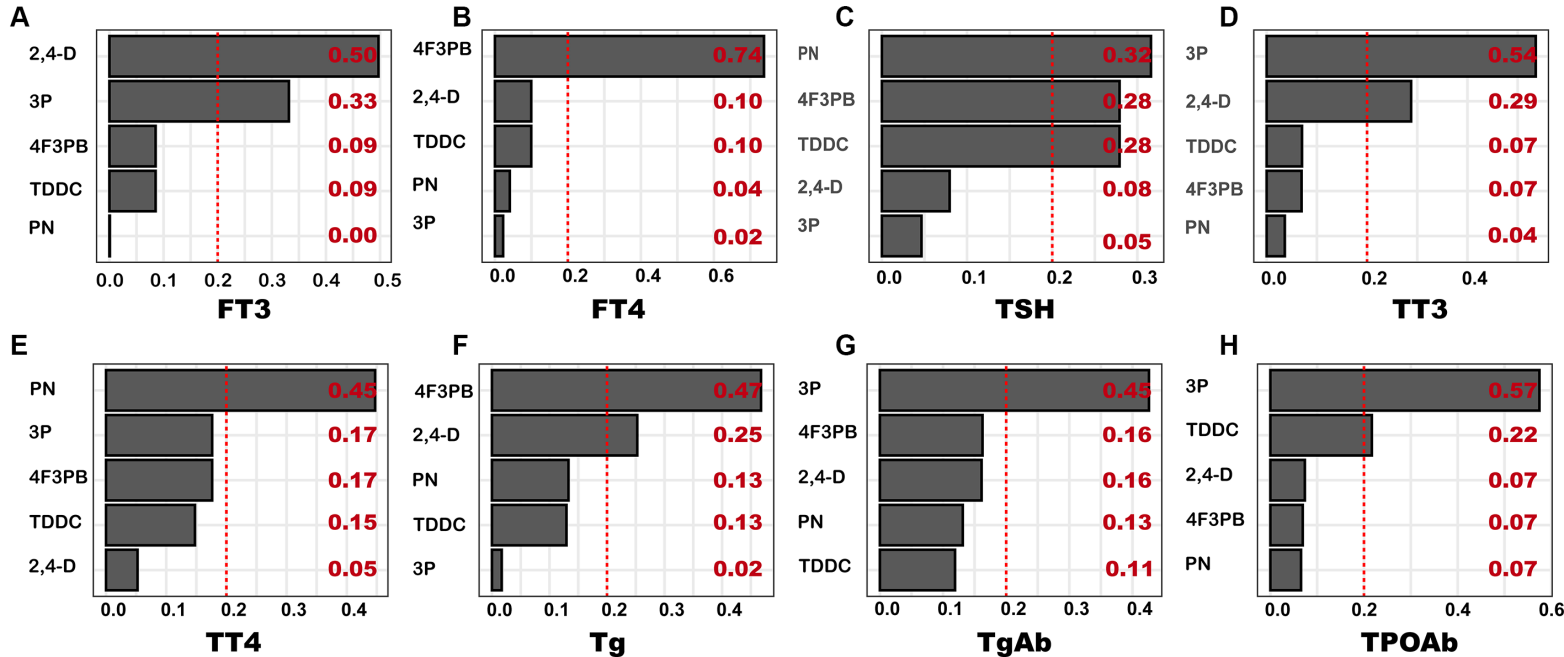
WQS-based weights of pesticide metabolites on thyroid function index. The model was adjusted for covariates, including age, sex, race, marital status, education, body mass index, smoking status, and alcohol consumption. **(A)** FT3; **(B)** FT4; **(C)** TSH; **(D)** TT3; **(E)** TT4; **(F)** Tg; **(G)** TgAb; **(H)** TPOAb.

The results of the relationship between the total WQS index and thyroid function indicators as well as the estimated chemical weight of each pesticide metabolite are provided in Figure 4. Based on the fully adjusted model, 2,4-D had the highest weight (0.50) on FT3, followed by 3P (0.33). Similarly, 4F3PB demonstrated the highest weight on FT4 (0.74); PN on TSH (0.32); 3P on TT3 (0.54); PN on TT4 (0.45); 4F3PB on Tg (0.47); 3P on TgAB (0.45); and 4F3PB on 3P (0.57).

## 4 Discussion

In this study, we initially revealed that pesticide exposure was negatively correlated with the thyroid function indexes FT4, TT4, TgAb, and TPOAb in patients with pesticide poisoning at our hospital. In a subsequent cross-sectional study of an adult population in the United States (NHANES 2007–2012), BKMR, linear regression, RCS, and WQS models were used to evaluate the individual and combined effects of pesticide metabolites on thyroid function indicators. In terms of the effects of each pesticide metabolite, positive correlations were found between 2,4-D and FT3 and between 4F3PB and FT4. Conversely, we observed a negative correlation of 4F3PB with Tg and TgAb, 3P with TgAb and TPOAb, PN with TSH and TT4, and TDDC with TPOAb.

In the case of multi-metabolic compounds, the BKMR model showed that the metabolic mixture of the different pesticides was negatively correlated with FT4, TSH, and Tg as well as with TgAB after the 60th percentile and TPOAb after the 70th percentile, consistent with the clinical data results of the patients in our hospital.

2,4-D is an extensively used herbicide, particularly in the United States and Canada. However, increasing concerns have been raised over its endocrine effects. Moreover, other studies have demonstrated that 2,4-D is an endocrine disruptor that may affect thyroid hormones in male but not in female rodents (8, 9). Similar sex-dependent findings were observed in humans, wherein a significant association was demonstrated between 2,4-D pesticide exposure and hypothyroidism in males, but no such association was detected in females (7). In our study, 2,4-D was positively correlated with FT3 in the general population, which persisted in the age group of ≥60 years. However, no other significant correlation was determined for the remaining thyroid function indicators, consistent with the US EPA findings. Nevertheless, the RCS dose–response curve revealed that thyroid function was affected at a 2,4-D dose exceeding a specific range, highlighting that the effect of the herbicide 2,4-D on the human body must not be overlooked.

In this study, we also analyzed the effects of the OP metabolites PN and TDDC on thyroid function indicators. TDDC is primarily a metabolite of dichlorphos, while PN is a metabolite of many OPs, including parathion and methyl parathion. Research has found that OPs can cause thyroid changes by affecting thyroid hormone regulation at the central nervous system level. In this mechanism, organophosphorus exposure leads to cholinergic overstimulation that further results in somatostatin activation, ultimately inhibiting TSH release (21). A cross-sectional study of adults and children in the United States found that the urinary organophosphorus metabolite 3,5,6-trichloro-2-pyridinol was positively correlated with serum total T4 and negatively associated with TSH in adolescents and adult males (22). Additionally, a rodent model investigation demonstrated that OP exposure could induce changes in thyroid hormone levels; however, the trends in the specific thyroid hormone levels were inconsistent. In particular, rodents that received oral doses of organophosphorus malathion exhibited reduced serum T4 and T3 levels and increased TSH concentrations compared with the mouse pups exposed to OPs *in utero* and postnatally (23). The above results are partially inconsistent with our current study findings, where we observed negative correlations of the OP metabolite PN with TSH and TT4 and that of TDDC with TPOAb. This discrepancy could be because different OP metabolites exert distinct effects on the thyroid gland. Moreover, the different results from the animal model study could be attributed to the variation in the metabolic pathways, species’ acceptance thresholds for OPs, or thyroid gland effects occurring during various developmental stages.

Additionally, we investigated the effects of the pyrethroid metabolites 3P and 4F3PB, which are more toxic than the mother compounds. The metabolites 3P and 4F3PB are among the most detectable substances in human urine and blood as well as animal tissues and thus are commonly used as biomarkers to assess pyrethroid exposure in humans (24–26). Studies on pyrethroid exposure in lizards have found that these pesticides and their metabolites 3P and 4F3PB have unique endocrine-disrupting mechanisms that can impair the thyroid system. Although metabolite exposure did not lead to any significant or slightly significant changes in the thyroid gland or thyroid hormone levels, significant alterations were observed in the expression of the thyroid axis-related genes in the liver and brain of the lizards (27). Several researchers have indicated that 3P has no significant effect on the human thyroid hormone (28), corroborating our current study results. However, apart from thyroid hormone examination, our study also assessed thyroid antibody and antibody protein levels. We demonstrated that although 3P was not significantly correlated with the thyroid hormone, it was negatively correlated with TgAb and TPOAb. Moreover, scarce research exists on the impact of pesticide exposure on these two indicators. Currently, relatively limited information is available on the effects of 4F3PB on thyroid function. The present study showed that 4F3PB was positively correlated with FT4 but negatively correlated with Tg and TgAB. All these thyroid function changes induced by pyrethroids and their metabolites may be ascribed to their structural similarity with the thyroid hormone receptors.

The previously mentioned findings represent individual analyses of the effects of each pesticide and its metabolites on thyroid function indicators. However, pesticide absorption by the human body does not encompass a single pesticide and generally involves two or more types of pesticides. Thus, we further analyzed the effects of pesticide mixtures on thyroid function indicators. A previous comparison study of the toxicity of four organophosphate pesticides (dichlorvos, dimethoate, acephate, and methamidophos) with that of a single pesticide in rats demonstrated that the toxicity of a single pesticide was weak or even non-toxic, but the combination of the four pesticides caused oxidative stress and liver and kidney dysfunction, disrupted lipid and amino acid metabolism, and interfered with thyroid function (29). This study analyzed the effects of OP, herbicide and pyrethroid mixtures on thyroid function in clinical patients in Binhai County People’s Hospital. The analyses revealed that the pesticide mixtures were negatively correlated with the thyroid function indicators FT4, TT4, TgAb, and TPOAb. Subsequently, in order to further validate the above findings, the effect of this pesticide mixture on thyroid function indicators was investigated in the NHANES database in the United States. The results showed that based on the BKMR model, the mixture was negatively correlated with FT4, TSH, Tg, and after the 60th percentile with TgAb and after the 70th percentile with TPOAb. These results were generally consistent with the clinical patient data from Binhai County People’s Hospital. Next, the weight of the effect of each metabolite in the pesticide mixture on thyroid function indicators was analyzed by the WQS model. Through the above findings, it was speculated that the alteration of thyroid function indicators might be caused by various thyroid damage mechanisms corresponding to each pesticide metabolite. However, the process by which these pesticides interact and cause thyroid damage is not known, and this area was not evaluated in our study. Nevertheless, this aspect is the direction of our future research. Despite these gaps in the literature, various pesticide mixtures have been established to cause damage to the thyroid gland. Thus, the potential harm from pesticide exposure during daily life, consumption of fruits and vegetables, and engagement in agricultural activities should not be disregarded, emphasizing the need to follow protective measures to reduce the health consequences on the human body.

Our study has several advantages that are worth mentioning. We used clinical data to analyze the relationship between patients with pesticide poisoning and thyroid function, along with the NHANES data for further evaluation. Moreover, the current study represents the first attempt to elucidate the link between herbicides, OPs, and pyrethroids (alone and combined) and thyroid function in United States adults. The study results indicated a potential relationship between these pesticides and thyroid function, suggesting the requirement for further mechanistic studies to explore the underlying physiological mechanisms. Another advantage is that we constructed WQS and BKMR models to examine the overall effects of the mixture of herbicides, OPs, and pyrethroids on thyroid function, followed by linear regression and RCS to determine the effects of each pesticide metabolite on thyroid function. These statistical strategies showed the frequency with which people were simultaneously exposed to multiple pesticides in real life. Additionally, combining the advantages and disadvantages of various pesticide approaches might help us better understand their mixed effects, ultimately enabling us to obtain more reliable conclusions and provide valuable scientific information on appropriate pesticide utilization. However, our study has certain limitations that should be acknowledged. The main limitation of this study is that a causal relationship between pesticides and thyroid function could not be confirmed due to the cross-sectional research design. Additionally, herbicides, OPs, and pyrethroids have shorter half-lives that range from a few hours to weeks; thus, the correlation of long-term exposure cannot be determined. Finally, the confounding effects of unmeasured factors cannot be ruled out from the study, even though adjustments were made for some thyroid function-related risk factors.

## 5 Conclusion

In this study, firstly, pesticides were found to be significantly negatively correlated with thyroid function indicators by analysing the data in the clinical patients in Binhai County People’s Hospital, and similarly, pesticide metabolites were found to have a significant difference on the thyroid function indicators in the NHANES database in the United States. Subsequently, through different statistical methods, it was found that there were significant differences in the effects of different metabolite weights on different thyroid function indicators. We initially discussed the effects of various pesticide metabolites on thyroid function indicators. At a later stage, we need to confirm our main findings with a large number of samples, further explore their effects on each thyroid disease, and elucidate the potential mechanisms between various pesticides and thyroid diseases.

## Data availability statement

The datasets presented in this study can be found in online repositories. The names of the repository/repositories and accession number(s) can be found at: https://wwwn.cdc.gov/nchs/nhanes/Default.aspx.

## Ethics statement

The studies involving humans were approved by Binhai County People’s Hospital. The studies were conducted in accordance with the local legislation and institutional requirements. The human samples used in this study were acquired from primarily isolated as part of your previous study for which ethical approval was obtained. Written informed consent for participation was not required from the participants or the participants’ legal guardians/next of kin in accordance with the national legislation and institutional requirements.

## Author contributions

LX: Conceptualization, Formal analysis, Methodology, Supervision, Writing – review & editing. SY: Data curation, Resources, Software, Writing – review & editing. LW: Project administration, Supervision, Writing – review & editing. JQ: Investigation, Project administration, Supervision, Writing – review & editing. HM: Project administration, Supervision, Writing – review & editing. LZ: Project administration, Supervision, Writing – review & editing. WS: Data curation, Software, Supervision, Validation, Writing – review & editing. AH: Conceptualization, Data curation, Investigation, Methodology, Resources, Software, Supervision, Validation, Visualization, Writing – original draft, Writing – review & editing.

## Footnotes

^1^https://www.cdc.gov/nchs/nhanes/irba98

^2^https://www.cdc.gov/nchs/data_access/restrictions

## References

[1] Axelstad M Boberg J Nellemann C Kiersgaard M Jacobsen PR Christiansen S. Exposure to the widely used fungicide mancozeb causes thyroid hormone disruption in rat dams but no behavioral effects in the offspring. Toxicol Sci. (2011) 120:439–46. doi: 10.1093/toxsci/kfr006 21266532

[2] Maranghi F De Angelis S Tassinari R Chiarotti F Lorenzetti S Moracci G. Reproductive toxicity and thyroid effects in Sprague Dawley rats exposed to low doses of ethylenethiourea. Food Chem Toxicol. (2013) 59:261–71. doi: 10.1016/j.fct.2013.05.048 23774258

[3] Panganiban L Cortes-Maramba N Dioquino C Suplido ML Ho H Francisco-Rivera A. Correlation between blood ethylenethiourea and thyroid gland disorders among banana plantation workers in the Philippines. Environ Health Perspect. (2004) 112:42–5. doi: 10.1289/ehp.6499 14698929 PMC1241795

[4] Du G Shen O Sun H Fei J Lu C Song L. Assessing hormone receptor activities of pyrethroid insecticides and their metabolites in reporter gene assays. Toxicol Sci. (2010) 116:58–66. doi: 10.1093/toxsci/kfq120 20410157

[5] Zúñiga-Venegas LA Hyland C Muñoz-Quezada MT Quirós-Alcalá L Butinof M Buralli R. Erratum: "Health effects of pesticide exposure in Latin American and the Caribbean populations: a scoping review". Environ Health Perspect. (2022) 131:89001. doi: 10.1289/EHP13645 PMC10434013 37589661

[6] Shrestha S Parks CG Goldner WS Kamel F Umbach DM Ward MH. Pesticide use and incident hypothyroidism in pesticide applicators in the agricultural health study. Environ Health Perspect. (2018) 126:97008. doi: 10.1289/EHP3194 30256155 PMC6375417

[7] Goldner WS Sandler DP Yu F Hoppin JA Kamel F Levan TD. Pesticide use and thyroid disease among women in the agricultural health study. Am J Epidemiol. (2010) 171:455–64. doi: 10.1093/aje/kwp404 20061368 PMC2842196

[8] Marty MS Neal BH Zablotny CL Yano BL Andrus AK Woolhiser MR. An F1-extended one-generation reproductive toxicity study in Crl:CD(SD) rats with 2,4-dichlorophenoxyacetic acid. Toxicol Sci. (2013) 136:527–47. doi: 10.1093/toxsci/kft213 24072463 PMC3858197

[9] Lacasaña M López-Flores I Rodríguez-Barranco M Aguilar-Garduño C Blanco-Muñoz J Pérez-Méndez O. Association between organophosphate pesticides exposure and thyroid hormones in floriculture workers. Toxicol Appl Pharmacol. (2010) 243:19–26. doi: 10.1016/j.taap.2009.11.008 19914268

[10] Campos É Freire C. Exposure to non-persistent pesticides and thyroid function: a systematic review of epidemiological evidence. Int J Hyg Environ Health. (2016) 219:481–97. doi: 10.1016/j.ijheh.2016.05.006 27265299

[11] Anadón A Martínez-Larrañaga MR Martínez MA. Use and abuse of pyrethrins and synthetic pyrethroids in veterinary medicine. Vet J. (2009) 182:7–20. doi: 10.1016/j.tvjl.2008.04.008 18539058

[12] Crofton KM. Thyroid disrupting chemicals: mechanisms and mixtures. Int J Androl. (2008) 31:209–23. doi: 10.1111/j.1365-2605.2007.00857.x 18217984

[13] Xu C Li X Jin M Sun X Niu L Lin C. Early life exposure of zebrafish (*Danio rerio*) to synthetic pyrethroids and their metabolites: a comparison of phenotypic and behavioral indicators and gene expression involved in the HPT axis and innate immune system. Environ Sci Pollut Res Int. (2018) 25:12992–3003. doi: 10.1007/s11356-018-1542-0 29480392

[14] Bravo R Caltabiano LM Weerasekera G Whitehead RD Fernandez C Needham LL. Measurement of dialkyl phosphate metabolites of organophosphorus pesticides in human urine using lyophilization with gas chromatography-tandem mass spectrometry and isotope dilution quantification. J Expo Anal Environ Epidemiol. (2004) 14:249–59. doi: 10.1038/sj.jea.7500322, PMID: 15141154

[15] Jayatilaka NK Restrepo P Williams L Ospina M Valentin-Blasini L Calafat AM. Quantification of three chlorinated dialkyl phosphates, diphenyl phosphate, 2,3,4,5-tetrabromobenzoic acid, and four other organophosphates in human urine by solid phase extraction-high performance liquid chromatography-tandem mass spectrometry. Anal Bioanal Chem. (2017) 409:1323–32. doi: 10.1007/s00216-016-0061-4 27838756 PMC5576356

[16] Bobb JF Claus Henn B Valeri L Coull BA. Statistical software for analyzing the health effects of multiple concurrent exposures via Bayesian kernel machine regression. Environ Health. (2018) 17:67. doi: 10.1186/s12940-018-0413-y 30126431 PMC6102907

[17] Bobb JF Valeri L Claus Henn B Christiani DC Wright RO Mazumdar M. Bayesian kernel machine regression for estimating the health effects of multi-pollutant mixtures. Biostatistics. (2015) 16:493–508. doi: 10.1093/biostatistics/kxu058 25532525 PMC5963470

[18] Keil AP Buckley JP O'Brien KM Ferguson KK Zhao S White AJ. A quantile-based g-computation approach to addressing the effects of exposure mixtures. Environ Health Perspect. (2020) 128:47004. doi: 10.1289/EHP5838 32255670 PMC7228100

[19] Coady K Marino T Thomas J Sosinski L Neal B Hammond L. An evaluation of 2,4-dichlorophenoxyacetic acid in the amphibian metamorphosis assay and the fish short-term reproduction assay. Ecotoxicol Environ Saf. (2013) 90:143–50. doi: 10.1016/j.ecoenv.2012.12.025 23357564

[20] Coady KK Kan HL Schisler MR Gollapudi BB Neal B Williams A. Evaluation of potential endocrine activity of 2,4-dichlorophenoxyacetic acid using in vitro assays. Toxicol In Vitro. (2014) 28:1018–25. doi: 10.1016/j.tiv.2014.04.006 24815817

[21] Phillips S Suarez-Torres J Checkoway H Lopez-Paredes D Gahagan S Suarez-Lopez JR. Acetylcholinesterase activity and thyroid hormone levels in Ecuadorian adolescents living in agricultural settings where organophosphate pesticides are used. Int J Hyg Environ Health. (2021) 233:113691. doi: 10.1016/j.ijheh.2021.113691 33581413 PMC7965258

[22] Fortenberry GZ Hu H Turyk M Barr DB Meeker JD. Association between urinary 3, 5, 6-trichloro-2-pyridinol, a metabolite of chlorpyrifos and chlorpyrifos-methyl, and serum T4 and TSH in NHANES 1999-2002. Sci Total Environ. (2012) 424:351–5. doi: 10.1016/j.scitotenv.2012.02.039, PMID: 22425279 PMC3327766

[23] Jeong SH Kim BY Kang HG Ku HO Cho JH. Effect of chlorpyrifos-methyl on steroid and thyroid hormones in rat F0- and F1-generations. Toxicology. (2006) 220:189–202. doi: 10.1016/j.tox.2006.01.005 16472551

[24] Barr DB Olsson AO Wong LY Udunka S Baker SE Whitehead RD. Urinary concentrations of metabolites of pyrethroid insecticides in the general U.S. population: National Health and nutrition examination survey 1999–2002. Environ Health Perspect. (2010) 118:742–8. doi: 10.1289/ehp.0901275, PMID: 20129874 PMC2898848

[25] Hwang M Lee Y Choi K Park C. Urinary 3-phenoxybenzoic acid levels and the association with thyroid hormones in adults: Korean National Environmental Health Survey 2012–2014. Sci Total Environ. (2019) 696:133920. doi: 10.1016/j.scitotenv.2019.133920 31446285

[26] Ratelle M Coté J Bouchard M. Time profiles and toxicokinetic parameters of key biomarkers of exposure to cypermethrin in orally exposed volunteers compared with previously available kinetic data following permethrin exposure. J Appl Toxicol. (2015) 35:1586–93. doi: 10.1002/jat.3124 25772368

[27] Chang J Pan Y Liu W Xu P Li W Wan B. Lambda-cyhalothrin and its common metabolite differentially modulate thyroid disruption effects in Chinese lizards (Eremias argus). Environ Pollut. (2021) 287:117322. doi: 10.1016/j.envpol.2021.117322 34000667

[28] Jain RB. Variability in the levels of 3-phenoxybenzoic acid by age, gender, and race/ethnicity for the period of 2001–2002 versus 2009–2010 and its association with thyroid function among general US population. Environ Sci Pollut Res Int. (2016) 23:6934–9. doi: 10.1007/s11356-015-5954-9, PMID: 26676543

[29] Du L Li S Qi L Hou Y Zeng Y Xu W. Metabonomic analysis of the joint toxic action of long-term low-level exposure to a mixture of four organophosphate pesticides in rat plasma. Mol BioSyst. (2014) 10:1153–61. doi: 10.1039/C4MB00044G 24626741

