# Supplementary material for: Association between pesticide exposure and thyroid function: analysis of Chinese and NHANES databases
Source: Front Public Health. 2024 Jun 12;12:1378027. doi: 10.3389/fpubh.2024.1378027 (PMC11210317; doi:10.3389/fpubh.2024.1378027)
Supplement: Supplementary file 1 [file Data_Sheet_1.docx]

Table S1 Detection method and reference value range of thyroid function index.

| Thyroid function (CHINA) | test method | standard value |
| --- | --- | --- |
| FT3 | Chemiluminescence immunoassay | 3.5-6.5Pmol/L |
| FT4 | Chemiluminescence immunoassay | 10-23Pmol/L |
| TSH | Chemiluminescence immunoassay | 2-10mU/L |
| TT3 | Chemiluminescence immunoassay | 0.92-2.79nmol/L |
| TT4 | Chemiluminescence immunoassay | 58.1-165.20nmol/L |
| TgAb | Chemiluminescence immunoassay | 0-60IU/ml |
| TPOAb | Chemiluminescence immunoassay | 0-60IU/ml |

| Thyroid function (NHANES) | test method | standard value |
| --- | --- | --- |
| FT3 | a competitive binding immunoenzymatic assay | 1.73-10.37pg/mL |
| FT4 | a two-step enzyme immunoassay | 0.43- 2.76ng/dL |
| TSH | two-site immunoenzymatic (“sandwich”) assay | 0.035- 55.49mIU/L |
| TT3 | a competitive binding immunoenzymatic assay | 50 - 288ng/dL |
| TT4 | a competitive binding immunoenzymatic assay | 2.9 -19.09μg/dL |
| TgAb | a sequential two-step immunoenzymatic "sandwich" assay | 0.07 - 395.21IU/mL |
| TPOAb | a sequential two-step immunoenzymatic "sandwich" assay | 0.6- 1635.1IU/mL |
