# Supplementary material for: Association between pesticide exposure and thyroid function: analysis of Chinese and NHANES databases
Source: Front Public Health. 2024 Jun 12;12:1378027. doi: 10.3389/fpubh.2024.1378027 (PMC11210317; doi:10.3389/fpubh.2024.1378027)
Supplement: Supplementary file 3 [file Data_Sheet_3.docx]

Table S3 Association between 2, 4-dichlorophenoxyacetic acid in herbicides and serum thyroid hormone in U.S. adults reported in the 2007-2012 NHANES study.

| Variables | 20-39 (n = 437) | p | 40-59 (n = 430) | p | ≥60 (n = 448) | p |
| --- | --- | --- | --- | --- | --- | --- |
| FT3 | 0.023(-0.017－0.062) | 0.254 | 0.014 (-0.002 -0.031) | 0.094 | **0.031 (0.002 -0.060)** | **0.039** |
| FT4 | -0.001(-0.009－0.009) | 0.965 | 0.001 (-0.005 -0.006) | 0.835 | 0.011 (-0.024 -0.045) | 0.540 |
| TSH | 0.006(-0.107－0.118) | 0.918 | -0.020 (-0.075 -0.035) | 0.475 | 0.675 (-0.699 -2.050) | 0.335 |
| TT3 | -0.444(-2.597－1.710) | 0.686 | 0.574 (-0.357 -1.505) | 0.226 | 1.898 (-0.204 -4.000) | 0.077 |
| TT4 | -0.039(-0.257－0.179) | 0.723 | -0.018 (-0.114 -0.078) | 0.713 | -0.060 (-0.294 -0.174) | 0.614 |
| Tg | -0.427(-1.199－0.344) | 0.277 | 0.191 (-0.842 -1.224) | 0.716 | -0.728 (-3.281 -1.825) | 0.576 |
| TgAb | -0.185(-0.432－0.062) | 0.141 | 1.575 (-1.596 -4.746) | 0.330 | -2.339 (-7.552 -2.874) | 0.378 |
| TPOAb | 1.263(-5.606－8.131) | 0.718 | -0.010 (-2.282 -2.261) | 0.993 | 4.736 (-8.635 -18.106) | 0.487 |

Table S4 Association between 4-fluoro-3-phenoxybenzoic acid in herbicides and serum thyroid hormone in U.S. adults reported in the 2007-2012 NHANES study.

| Variables | 20-39 (n = 437) | p | 40-59 (n = 430) | p | ≥60 (n = 448) | p |
| --- | --- | --- | --- | --- | --- | --- |
| FT3 | 0.083(-0.556－0.723) | 0.798 | -0.158 (-0.590 -0.273) | 0.471 | **-0.205 (-0.310**－**-0.099)** | **＜0.001** |
| FT4 | -0.062(-0.152－0.028) | 0.179 | **0.238 (0.031 -0.445)** | **0.024** | **0.110 (0.083**－**0.137)** | **＜0.001** |
| TSH | -0.533(-1.553－0.487) | 0.305 | **-1.257 (-1.967**－**-0.547)** | **0.001** | 0.816 (-0.593 -2.225) | 0.256 |
| TT3 | 3.217(-24.553－30.988) | 0.820 | -8.841 (-27.386－-9.704) | 0.349 | **-11.858 (-15.205**－**-8.512)** | **＜0.001** |
| TT4 | -0.401(-1.378－0.576) | 0.420 | 1.178 (-0.835 -3.191) | 0.251 | **0.887 (0.599**－**1.174)** | **＜0.001** |
| Tg | **-15.798(-22.855－-8.741)** | **＜0.001** | -1.810 (-16.815 -13.194) | 0.813 | **-11.498 (-16.314**－**-6.682)** | **＜0.001** |
| TgAb | **-1.752(-3.469－-0.034)** | **0.046** | -5.226 (-15.862 -5.409) | 0.335 | **-14.616 (-24.594**－**-4.637)** | **0.004** |
| TPOAb | 33.180(-63.517－129.877) | 0.500 | **-36.148 (-61.467**－**-10.830)** | **0.005** | **-12.769 (-19.364**－**-6.173)** | **＜0.001** |

Table S5 Association between 3-phenoxybenzoic acid in herbicides and serum thyroid hormone in U.S. adults reported in the 2007-2012 NHANES study.

| Variables | 20-39 (n = 437) | p | 40-59 (n = 430) | p | ≥60 (n = 448) | p |
| --- | --- | --- | --- | --- | --- | --- |
| FT3 | 0.006(-0.001－0.0127) | 0.103 | -0.005 (-0.014 -0.005) | 0.349 | 0.005 (-0.016 -0.025) | 0.660 |
| FT4 | 0.002(-0.001－0.004) | 0.121 | 0.002 (-0.003 -0.006) | 0.399 | -0.002 (-0.009 -0.005) | 0.555 |
| TSH | 0.007(-0.018－0.032) | 0.607 | **-0.039 (-0.070**－**-0.008)** | **0.014** | -0.032 (-0.135 -0.072) | 0.549 |
| TT3 | -0.104(-0.628－0.421) | 0.697 | -0.333 (-1.066 -0.401) | 0.373 | 0.398 (-0.894 -1.691) | 0.545 |
| TT4 | 0.003(-0.026－0.032) | 0.848 | -0.009 (-0.069 -0.052) | 0.779 | 0.034 (-0.057 -0.125) | 0.465 |
| Tg | 1.168(-0.794－3.129) | 0.243 | 0.201 (-0.168 -0.569) | 0.285 | -0.020 (-3.298 -3.258) | 0.991 |
| TgAb | 0.023(-0.096－0.142) | 0.701 | **-0.398 (-0.783**－**-0.013)** | **0.043** | -0.828 (-4.050 -2.394) | 0.614 |
| TPOAb | 0.278(-1.029－1.585) | 0.676 | **-2.046 (-3.124**－**-0.967)** | **＜0.001** | -0.257 (-3.344 -2.829) | 0.870 |

Table S6 Association between para-Nitrophenol in herbicides and serum thyroid hormone in U.S. adults reported in the 2007-2012 NHANES study.

| Variables | 20-39 (n = 437) | p | 40-59 (n = 430) | p | ≥60 (n = 448) | p |
| --- | --- | --- | --- | --- | --- | --- |
| FT3 | -0.002(-0.026－0.022) | 0.849 | -0.004 (-0.025 -0.017) | 0.720 | -0.015 (-0.034 -0.004) | 0.131 |
| FT4 | -0.002(-0.003－0.001) | 0.053 | -0.007 (-0.016 -0.001) | 0.086 | 0.001 (-0.006 -0.007) | 0.864 |
| TSH | **-0.032(-0.051－-0.014)** | **0.001** | **-0.103 (-0.188－-0.018)** | **0.017** | -0.013 (-0.083 -0.057) | 0.718 |
| TT3 | -0.207(-0.809－0.395) | 0.500 | -0.297 (-1.646 -1.051) | 0.665 | **-0.940 (-1.806－-0.074)** | **0.033** |
| TT4 | -0.041(-0.097－0.014) | 0.143 | -0.071 (-0.155 -0.012) | 0.093 | -0.019 (-0.079 -0.041) | 0.531 |
| Tg | -0.149(-0.389－0.090) | 0.221 | 0.652 (-0.878 -2.181) | 0.403 | 0.961 (-0.891 -2.813) | 0.308 |
| TgAb | -0.053(-0.107－0.002) | 0.058 | -0.171 (-1.470 -1.127) | 0.796 | 4.779 (-4.928 -14.487) | 0.334 |
| TPOAb | **-0.858(-1.699－-0.017)** | **0.046** | 0.145 (-4.312 -4.601) | 0.949 | -0.690 (-1.947 -0.567) | 0.281 |

Table S7 Association between trans-dichlorovinyl dimethylcyclopropane carboxylic acid in herbicides and serum thyroid hormone in U.S. adults reported in the 2007-2012 NHANES study.

| Variables | 20-39 (n = 437) | p | 40-59 (n = 430) | p | ≥60 (n = 448) | p |
| --- | --- | --- | --- | --- | --- | --- |
| FT3 | 0.004(-0.005－0.013) | 0.384 | 0.001 (-0.009 -0.009) | 0.996 | 0.001 (-0.012 -0.013) | 0.963 |
| FT4 | -0.001(-0.004－0.004) | 0.963 | 0.002 (-0.001 -0.006) | 0.210 | 0.001 (-0.003 -0.006) | 0.636 |
| TSH | 0.007(-0.036－0.050) | 0.740 | **-0.049 (-0.079**－**-0.019)** | **0.001** | -0.011 (-0.095 -0.072) | 0.788 |
| TT3 | 0.079(-0.902－1.061) | 0.874 | -0.367 (-0.813 -0.080) | 0.108 | 0.096 (-0.652 -0.845) | 0.800 |
| TT4 | -0.016(-0.055－0.023) | 0.427 | 0.005 (-0.039 -0.050) | 0.819 | 0.019 (-0.039 -0.077) | 0.517 |
| Tg | 1.896(-1.897－5.690) | 0.326 | 0.127 (-0.304 -0.559) | 0.562 | 0.329 (-1.622 -2.281) | 0.740 |
| TgAb | -0.062(-0.133－0.010) | 0.090 | -0.480 (-1.007 -0.046) | 0.074 | 0.370 (-3.194 -3.935) | 0.838 |
| TPOAb | -0.360(-1.747－1.027) | 0.610 | **-2.517 (-3.823**－**-1.210)** | **0.000** | **-1.009 (-1.942**－**-0.077)** | **0.034** |
